# Descriptive embryological insights of the colorectum of quail embryos with concern to its functional morphology

**DOI:** 10.1186/s12917-024-04341-z

**Published:** 2024-11-06

**Authors:** Fatma Abdelhakeem, Fatma A. Madkour

**Affiliations:** Department of Anatomy and Embryology, South Valley University, Qena, 83523 Egypt

**Keywords:** Colorectum, Myenteric plexus, Quail, Rectal glands, Telocytes

## Abstract

**Background:**

Quail is an interesting emerging bird species gaining attention in developmental embryology research due to its small size, quick lifespan, and fast growth rate. These characteristics make quail an ideal model for examining the development of the gastrointestinal tract. Consequently, the embryonic development of the colorectum was conducted to provide a comprehensive understanding of its functions in digestion, absorption, and immunity.

**Methodology:**

The morphological anatomy and microscopical structure of the colorectal wall of 74 embryos were studied using light and scanning electron microscopy (SEM). Histologically, the embryos were collected and dissected to extract the intestine. The samples were then fixed in 10% neutral buffer formalin for a minimum of 24 h, and in 2.5% glutaraldehyde buffer formalin for semithin processing and scanning electron microscopy.

**Results:**

The wall of the embryonic colorectum on the hatching day consisted of three layers; mucosa, muscularis externa, and serosa. Mucosa was a simple layer of columnar enterocytes interspersed with goblet cells that appeared as cub-like shaped cells. Additionally, two ganglionic plexuses were also developed in the colorectal wall; Auerbach plexus (among the colorectal tunica muscularis) and Meissner plexus (submucosal plexus).

**Conclusion:**

The morphological characteristics of the quail colorectum at different ages were closely related to its functional features.

## Introduction

The large intestine consists of two main parts: the colorectum and two caeca [1, 2]. The rectal wall is structured with four tunics; mucosa, submucosa, muscularis, and serosa. The mucosa consists of numerous villi, which are lined by simple columnar epithelium studded with goblet cells, and the muscular layer is formed of inner circular and outer longitudinal muscle fibers. Furthermore, the serosa is a single layer of squamous cells [3, 4]. It is worth to mention that the gastrointestinal tract (GIT) has two types of innervations; intrinsic enteric neurons and extrinsic efferent, and afferent nerves. The intrinsic enteric nervous system (ENS) innervates most gut regions, and consists of two ganglionic plexuses; the myenteric and submucosal ganglia. These plexuses contain various types of neurons and glial cells that arise from vagal neural crest cells and play a crucial role in regulating the function of the GIT [5]. The morphology of avian rectum is adapted to perform different specialized functions as fiber digestion with the symbiotic bacteria [6], absorption of water, Na^+^ [7, 8], folic acid [9], maintaining the bird weight during flying through eliminating the undesirable food, absorbing nutrients, water effectively, and protecting the lining mucosa [4].

The term evolutionary developmental biology, or EvoDevo, refers to the new morphological traits that enable the adoption of a new function in response to environmental conditions [10] and it recently proved the relation of evolutionary developmental biology and functional morphology [11]. Embryonic period is a very sensitive and critical stage during the development of the offspring’s phenotype [12]. Overall, quail is regarded as the perfect animal model utilized in developmental biology [13], these birds mature rapidly, have short incubation periods, and are easy to breed in a laboratory [14]. Research on the development of the colorectum is uncommon. Thus, the current study aims to enhance our understanding of the microscopic structure, morphology, and morphometric parameters of the embryonic colorectum in quail during the prehatching period. Importantly, this study contributes to a series of investigations focused on the microscopic structure of the intestinal tract during this developmental stage [15, 16].

## Materials and methods

### Sample collection and preparation

This study was conducted on seventy-four fertile eggs. These eggs obtained from South Valley University’s Japanese quail farm in the Qena governorate, Egypt. The fertile eggs were incubated at 37 °C and 55% relative humidity, according to Abdelhakeem [15]. Subsequently, the eggs were cracked open from the wide end, and the embryos were harvested from the third day of development until they hatched. The embryos were removed, cleaned with saline solution (0.9% NaCl), and fixed in neutral buffered formalin for paraffin-embedded sectioning, gross morphology, and 2.5% glutaraldehyde (pH 7.4) for ultrathin sectioning and SEM.

### Paraffin block technique

Three samples were collected for each age stage (3, 4, 5, 6, 8, 9, 10, 11, 12, 13, 15, and 17 days). For the early ages, the entire embryo was used, while for the older ages the colorectum was dissected from the surrounding viscera and excised for the preparation of paraffin blocks. The specimens were washed with saline, fixed in neutral buffer formalin (10%), dehydrated using ethyl alcohol (70%, 80%, 90%, and 100%), cleared in methyl benzoate, and finally coated with paraffin wax.

The paraffin blocks were cut and sectioned serially at a thickness 5 μm through a semi-automated sliding microtome. The sections were fixed on glass slides, dried, deparaffinized, rehydrated, and then stained with Harris’s Hematoxylin and Eosin (H&E), Mallory trichrome, methylene blue, Periodic Acid Schiff (PAS), and Alcian blue (AB) stains. The latter staining techniques were in accordance with the methods of the authors [17, 18]. The stained slides were examined, analyzed, and photographed using a DMLS light microscope (Leica, Germany) equipped with an MC120 HD camera.

### Resin block technique

Two age stages were subjected to semithin sectioning (three embryos from the12th, and 17th days of incubation). The colorectum samples were washed with phosphate buffered saline, and fixed in glutaraldehyde (2.5%) at 4 °C and then in osmic acid (1%) [19]. The specimens were dehydrated in ascending acetone grades before being implanted in acetone-resin mix and pure resin. Resin blocks were polymerized in an oven for two to three days at 60 °C. Semithin Sect. (1 μm) were prepared using an ultramicrotome (Ultracut E, Reichert-Leica, Germany) and stained with either methylene blue or toluidine blue.

### Scanning electron microscopy (SEM)

Three embryos, aged 12 and 17 days of incubation, were subjected to scanning electron microscopy. After the colorectum was eviscerated from the body cavity, it was thoroughly rinsed with saline and cut into three sections; one section was opened longitudinally at both ends. The samples were fixed for 24 h at 4 °C in 2.5% glutaraldehyde (pH 7.4), rinsed three times for 15 min in phosphate buffer solution (PBS) (pH 7.4), and postfixed for one hour at 4 °C using 1% osmium tetroxide (OsO4). Subsequently, the samples were subjected to four rounds of 15 min of PBS washing and rinsing, followed by dehydration using a graded sequence of alcohol concentrations (30, 50, 70, 95, and 100%), 30 min for each grade and two days for 100% concentration. After two days of post-fixation of the dehydrated samples in isoamyl acetate, the samples were dried and coated with gold. The specimens were examined and imaged using a JEOL scanning electron microscope (JSM-5400, Japan) at an accelerating voltage of 8 kV. This analysis took place in the electron microscopy unit at South Valley University’s central laboratory in Qena, Egypt.

### Gross morphology

Five embryos from the 9th, 11th, 12th, 13th, 15th, and 17th days of incubation were employed for gross anatomy and morphometrical measurements. The colorectum and two caeca were carefully dissected, removed from the body cavity, and then photographed.

### Morphometric measurements

The micro-morphometric measurements of the colorectal layers of each age-stage: Height of villi, number of villi, thickness of muscle layer, diameter of colorectal lumen, and diameter of colorectum were taken using Image J software (http://Fiji.sc/Fiji). All measurements were expressed (µm) in Table 1.

**Table 1:** Different histomorphometrical measurements of the quail rectum on 5, 9, 12, 15 and 17 days of incubation

Age of embryo	Diameter of colorectum (µm)	Diameter of colorectum lumen (µm)	Thickness of muscle layer (µm)	Number of villi	Height of villi (µm)
5d	178.98 ± 1.61 ^a^	27.70 ± 3.82 ^a^	-	4 ^a^	43.56 ± 3.27
9d	398.70 ± 3.77 ^b^	62.78 ± 1.11 ^b^	57.08 ± 6.89 ^a^	10 ^b^	42.09 ± 2.13 ^a^
12d	789.97 ± 8.55 ^c^	453.07 ± 1.24 ^c^	93.91 ± 1.78 ^b^	35 ^c^	37.81 ± 1.80 ^a^
15d	967.33 ± 6.65 ^d^	704.36 ± 2.34 ^d^	103.07 ± 8.21 ^b^	56 ^d^	36.90 ± 8.21 ^a^
17d	1185.00 ± 2.66 ^e^	610.28 ± 9.01 ^e^	197.03 ± 4.87 ^c^	102 ^e^	68.43 ± 8.87 ^b^

Gross morphometric measurements were taken in millimeters using precision digital Vernier caliper: including length of intestine, large intestine, rectum, and caeca. All data are recorded in Table 2.

**Table 2:** Different macroscopic measurements of the quail intestine and its parts on 11, 13, 15 and 17 days of incubation

Age of embryo	Total length of Intestine (mm)	Length of large intestine (mm)	Length of ceca (mm)	Length of rectum (mm)
E 11	66.74 ± 2.38	11.88 ± 0.83	6.13 ± 0.55	5.74 ± 0.66
E 13	95.65 ± 7.18	19.61 ± 2.32	10.89 ± 1.56	8.72 ± 0.75
E15	106.8 ± 3.41	23.90 ± 1.66	13.01 ± 0.55	10.89 ± 1.38
E17	138.43 ± 4.47	31.84 ± 1.47	16.36 ± 0.87	15.48 ± 0.95

### Statistical analysis

The data were statistically analyzed using IBM SPSS Software (version 22) and presented as Mean ± SD. Statistical differences between different age groups were measured by one-way ANOVA test followed by Bonferroni post-hoc test. P value < 0.05 was considered significant.

### Ethics statement

The current study was carried out at the Faculty of Veterinary Medicine, South Valley University, Qena, Egypt, following Egyptian animal law and the guiding principles of the institutional ethical council [Authorization number: VM/SVU/23(2)-07]. The study’s application complied with the ARRIVE guidelines.

The collection of embryos and all experimental protocols were conducted in accordance with relevant guidelines and regulations approved by the local ethical committee. All national and institutional guidelines for animal care and use were followed throughout this study. The embryos used in this research were obtained from the Japanese quail farm at South Valley University, Qena, Egypt.

## Results

### Microscopic observations

**Fig. 1:**
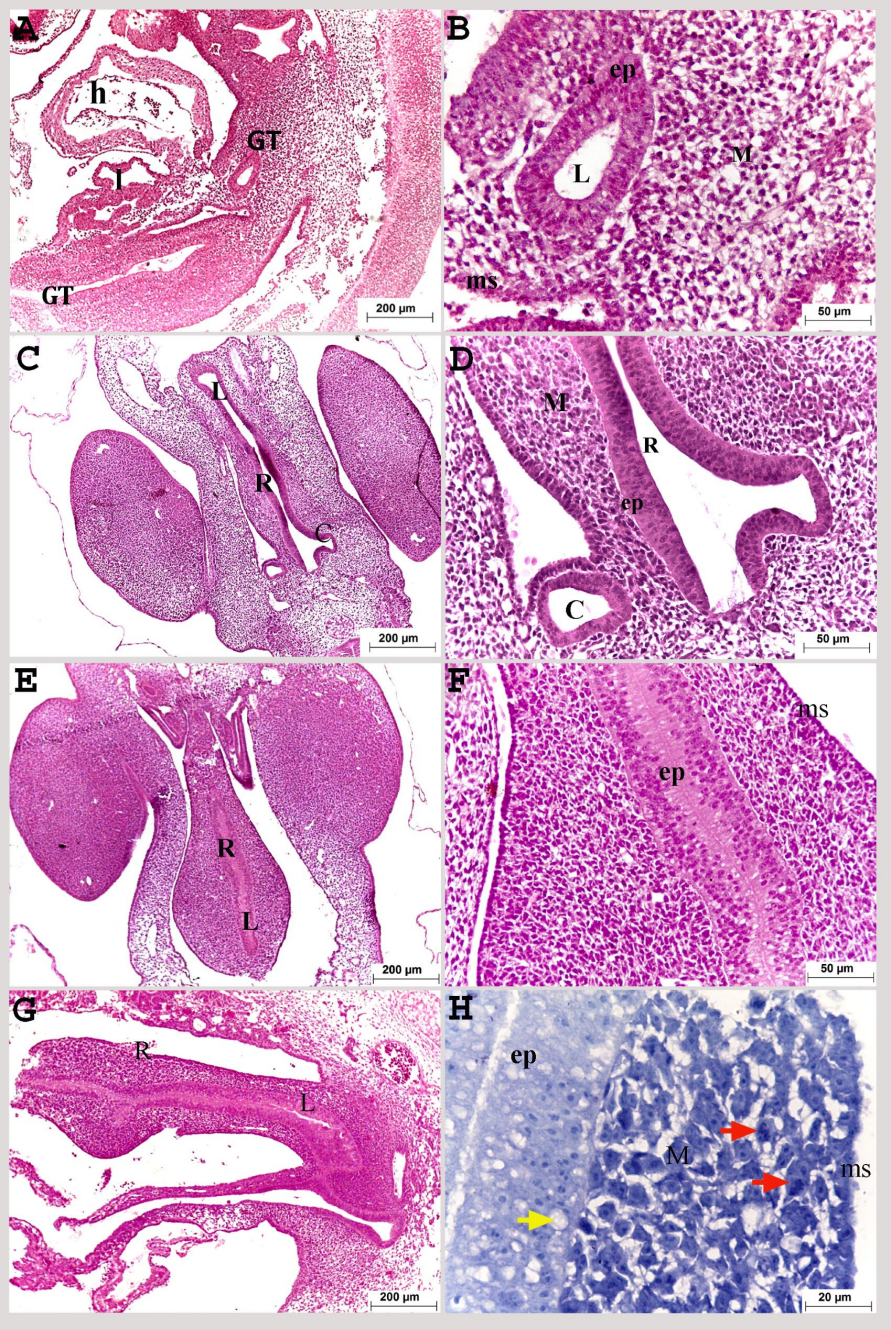
Colorectum of a quail embryo at day 3 (**A**, **B**) and 4 of incubation (**C**-**H**). Histomorphology. Longitudinal (**A**, **B**, **G**, **H**) and cross paraffin sections (**C**-**F**). H&E (**A**–**G**) and methylene blue stains (**H**). Images B, D, F and H were details of images A, C, E, G. Gut tube (GT), heart (h), liver (l), colorectum (R), caecum (C), lumen of colorectum (L), pseudostratified epithelium (ep), vacuoles (yellow arrow), mesenchyme (M), mitotic division (red arrow) and mesothelium (ms)

The gut tube was distinguished on the third day of incubation, and connected ventrally to the embryonic heart and liver. It was composed mainly of pseudostratified endoderm, mesoderm, and stratified mesothelium that consisted of two or three cuboidal cell layers (Fig. 1A, B).

On the fourth day of incubation, the hindgut occupied the most caudal part of the body cavity which distinguished from the rest of the gut tube by the emergence of the cecal primordia. It was related dorsally to the embryonic kidney, and ventrally to the body wall. The rectum (the colorectum) was represented the most caudal part of the hindgut, connected cranially to the two caeca which was enlarged like a balloon and tapered caudally. Some rectal parts, especially the most caudal part, still had obliterated lumen while others had canalized lumen (Fig. 1E, G). The wall was formed of pseudostratified epithelium and a simple cuboidal layer of mesothelium. The mesenchymal cells in between were characterized by high mitotic division (Fig. 1C: H). Extracellular vacuoles appeared within the pseudostratified epithelium (Fig. 1H).

**Fig. 2:**
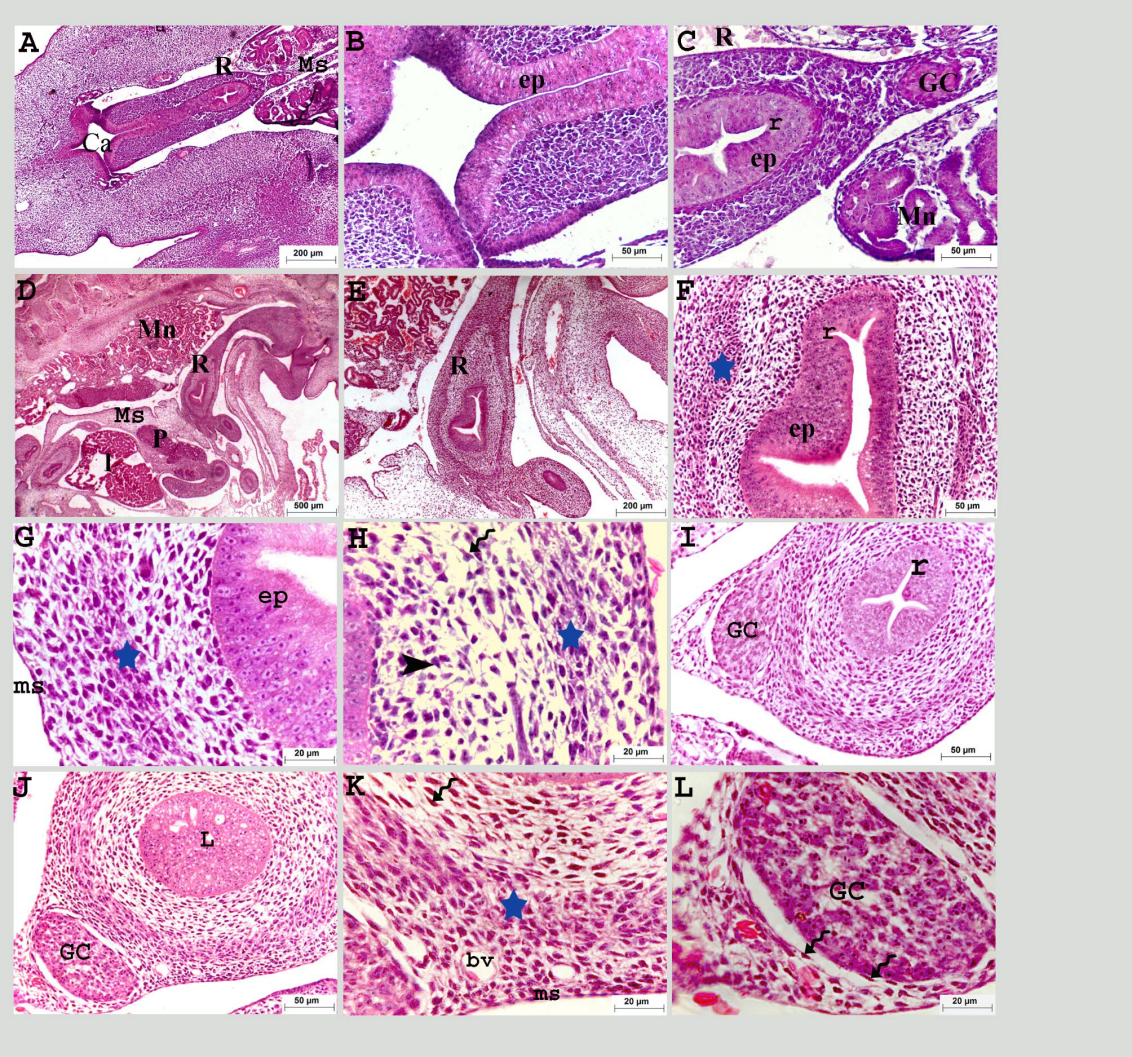
Colorectum of a quail embryo at day 5 (**A**-**C**) and 6 of incubation (**D**-**L**). Histomorphology. Cross (**A**-**C**, **I**-**L**) and longitudinal paraffin sections (**D**-**F**). H&E stain (**A**–**I**). Images B, C, E-I, K, and L were details of images A, D and J. Colorectum (R), cloaca (Ca), mesentery (Ms), pancreas (P), liver (l), mesonephros (Mn), pseudostratified epithelium (ep), previllous ridges (r), ganglionic cells (GC), mesenchymal condensation (star), telocytes (twisted arrows), mesenchymal cells (black arrow head) and mesothelium (ms), obliterated lumen (L), and blood vessels (bv)

The rectum was hung dorsally to the roof of the caudal body cavity with the mesentery and continued ventrally with the cloaca (Fig. 2A, B). On the fifth day of incubation, four invaginations were observed through the pseudostratified epithelium, forming previllous ridges projecting into the intestinal lumen. Ganglionic cell aggregation was observed within the mesenchyme at the level of the mesentery. These ganglionic cells were the beginning of development for the enteric nerve system (ENS) (Fig. 2A, C).

On the sixth day of incubation, the rectum continued caudally to open into the cloaca ventral to the mesonephros and gonads, the part closest to the cloaca was still obliterated (Fig. 2D, E). The condensed layer of mesenchymal cells was detected within the mesenchyme as the prospective muscular layer. Different cell types were recognized within the lamina propria: telocytes and mesenchymal cells. The telocyte had a small body and prolonged telopodes, surrounding the ganglionic cells (Fig. 2F: L). The mesothelium transformed into a simple layer of flattened cells (Fig. 2G, H, K).

**Fig. 3:**
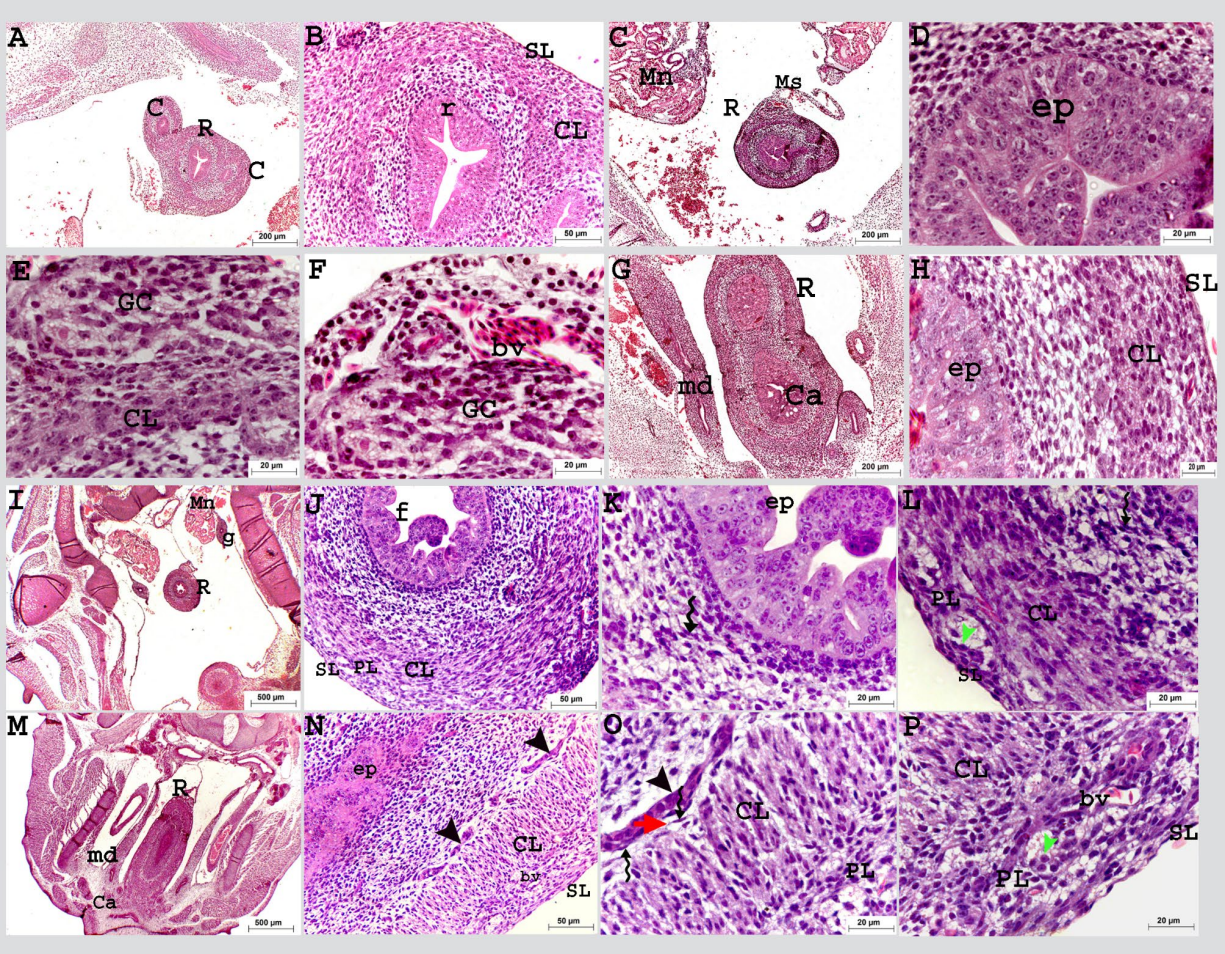
Colorectum of a quail embryo at day 8 (**A-H**) and 9 of incubation (**I-P**). Histomorphology. Longitudinal (**A**, **B**) and cross paraffin sections (**C-P**). H&E stain (**A–P**). Images B, D-F, H, J-L, K and N-P were details of images A, C, G, I and M. Colorectum (R), caecum (C), mesonephros (Mn), mesentery (Ms), previllous ridge (r), mesonephric duct (md), pseudostratified epithelium (ep), epithelium folds (f), circular muscular layer (CL), solitary groups of myoblasts (black arrow head), ganglionic cells (GC), cloaca (Ca), gonad (g), telocytes (twisted arrows), telopodes (red arrow head), myenteric plexus (PL) consisted of nerve cell bodies (green arrow head), blood vessel (bv), and serosa (SL)

On the eighth day of incubation, at the level of the two caeca, the rectal epithelium had four folds while caudally, the epithelial folds increased in number and occluded the lumen. Further at the level where it opened to the cloaca, the rectum was still uncanalized (Fig. 3A: C, G). The prospective muscular layer was more developed, increased in thickness and consisted of myoblasts. At the level with the two caeca, this layer continued with the caecal circular muscular layer (Fig. 3B, E, H).

On the ninth day of incubation, the rectum was situated in the middle of the roof of the caudal part of the body cavity, ventral to the mesonephros, and close to its final end, where it opened into the cloaca. It was also situated ventrally in the floor of the body cavity and the lumen of the caudal part was still obliterated until this age (Fig. 3I, M). The inner circular smooth muscular layer was completely developed and consisted of mature myocytes surrounded by network of telocyte. Solitary aggregated myoblasts were recognized above the circular layer (Fig. 3J, L, N: P). The myenteric plexus (Auerbach’s plexus) was one of the enteric nerve systems. It was recognized below the inner circular smooth muscle layer on the 9th day of incubation. The myenteric plexus appeared as connective tissue capsules surrounding groups of nerve cell bodies (Fig. 3L, O, P).

**Fig. 4:**
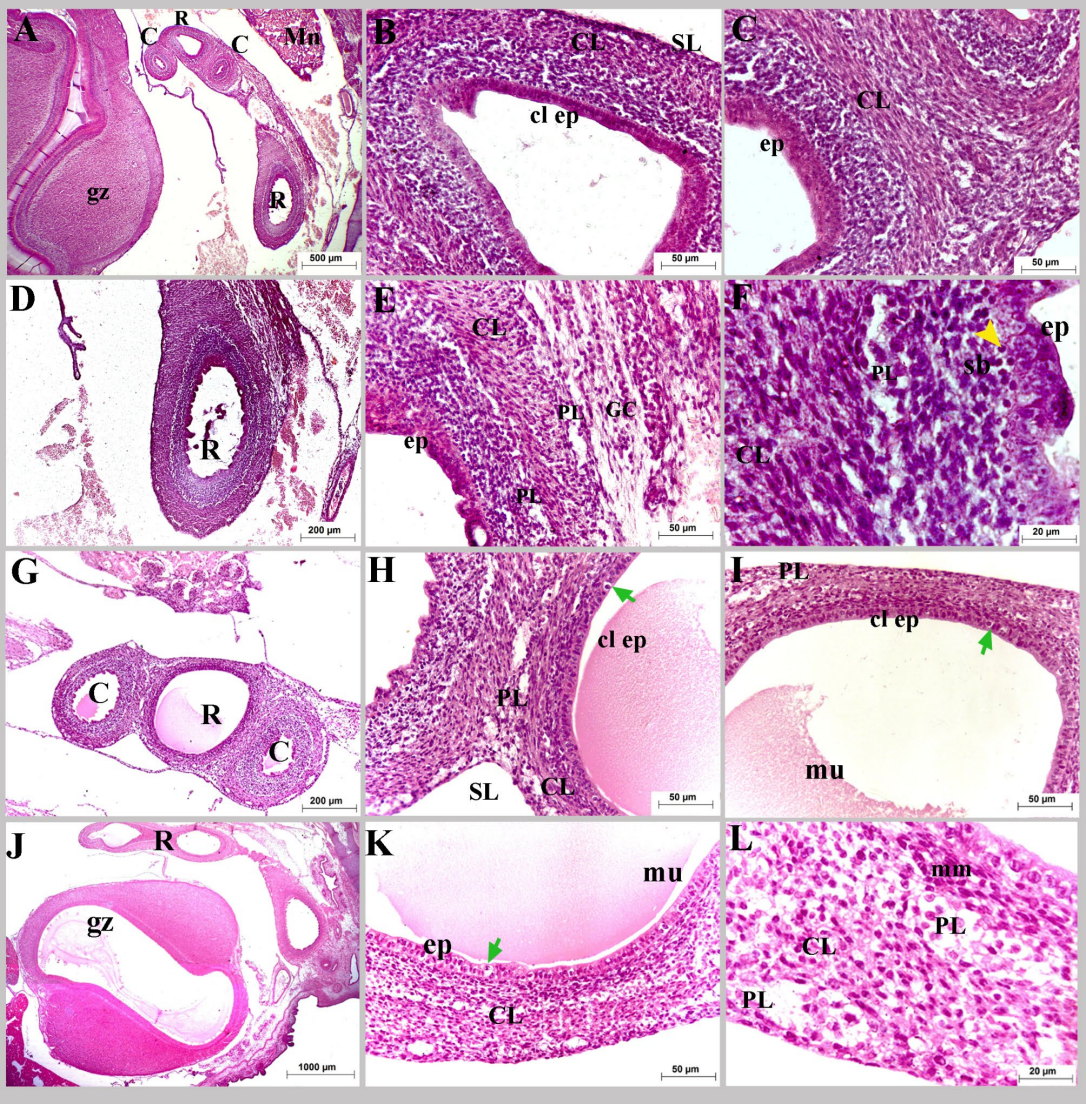
Colorectum of a quail embryo at day 10 (**A-F**) and 11 of incubation (**G-L**). Histomorphology. Longitudinal paraffin sections (**A-L**). H&E stain (**A-L**). Images B, C, E, F, H, I, K and L were details of images A, D, G and J. Colorectum (R), caeca (C), gizzard (gz), mesonephros (Mn), pseudostratified epithelium (ep), simple columnar epithelium (cl ep), enteroendocrine cell (green arrow), muscularis mucosa (mm), circular muscular layer (CL), ganglionic cells (GC), myenteric plexus (PL), mucous secretion (mu), and serosa (SL)

On the tenth day of incubation, the colorectum crossed caudoventrally in the dorso-caudal position to the gizzard in the roof of the body cavity (Fig. 4A). The epithelial folds disappeared and changed into a flat epithelium; a simple columnar type in some parts, leaving the colorectum with a wide lumen in comparison to that of the previous ages. At this time, the colorectum was completely canalized (Fig. 4B: F).

On the eleventh day of incubation, the colorectal wall epithelium was completely differentiated into simple columnar type; some large cells were almost along the entire length of the colorectum, these cells had large round nuclei and faint cytoplasm detected among columnar cells called enteroendocrine cells. At this age, the submucosal (Meissner) plexus was recognized between the mucosa and muscularis. It had the same structure as the myenteric plexus. The myenteric (Auerbach’s) plexus surrounding the circular muscular layer formed a network with those of the two caeca (Fig. 4G: M).

**Fig. 5:**
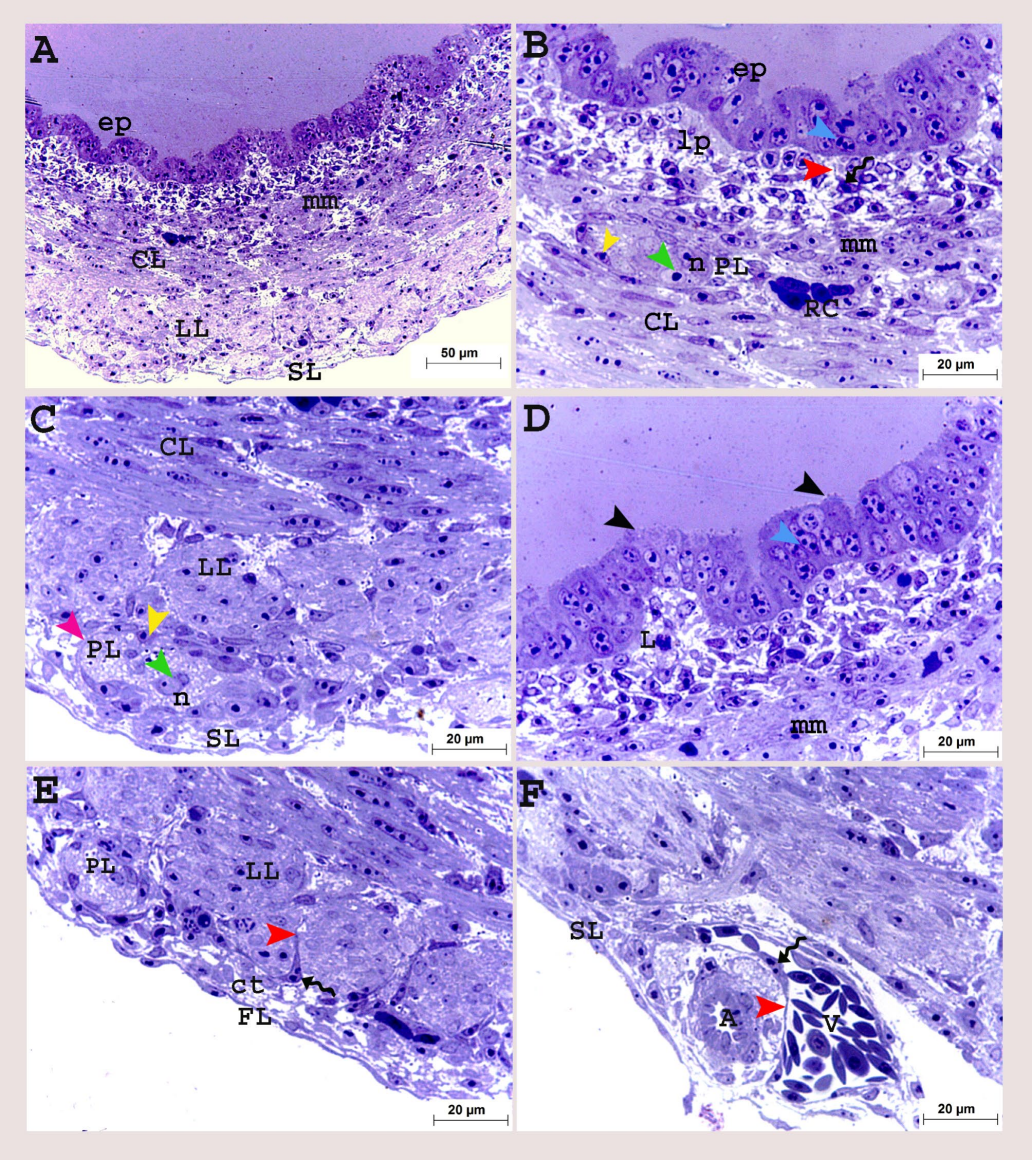
Colorectum of a quail embryo at day 12 of incubation (**A-F**). Histomorphology. Cross semithin sections (**A-F**). Toluidine blue stain (**A-F**). Images B-F were details of image A. Pseudostratified epithelium (ep), epithelium budding (black arrow head), lamina propria (lp), lymphocyte (L), mitotic divisions (blue arrow head), muscularis mucosa (mm), circular (CL), longitudinal (LL) muscular layers surrounded with telocytes (twisted arrows), telopodes (red arrow head), myenteric plexus (PL) consisted of capsule (purple arrow head) contained nerve cell bodies (n and green arrow head) flattened cells (yellow arrow head), artery (A), vein (V), red blood cell (RC), serosa (SL) consisted of connective tissue layer (ct) and layer of flattened cells (FL)

On the twelfth day of incubation, the epithelium began to fold forming villi, the enterocytes were in mitotic divisions and the upper border showed budding activity. The muscularis mucosa was differentiated as a thin layer of circular smooth muscle cells underneath the surface epithelium (Fig. 5A, B, D). The outer longitudinal muscular layer was developed below the circular layer, surrounded by telocytes. These cells had large bodies with long processes (telopodes). The myenteric plexus was observed below the longitudinal muscular layer and another type of cells recognized with neurons, supporting glial cells. The serosa consisted of a thin flattened cell layer with abundant connective tissue (Fig. 5A, C, E, F).

**Fig. 6:**
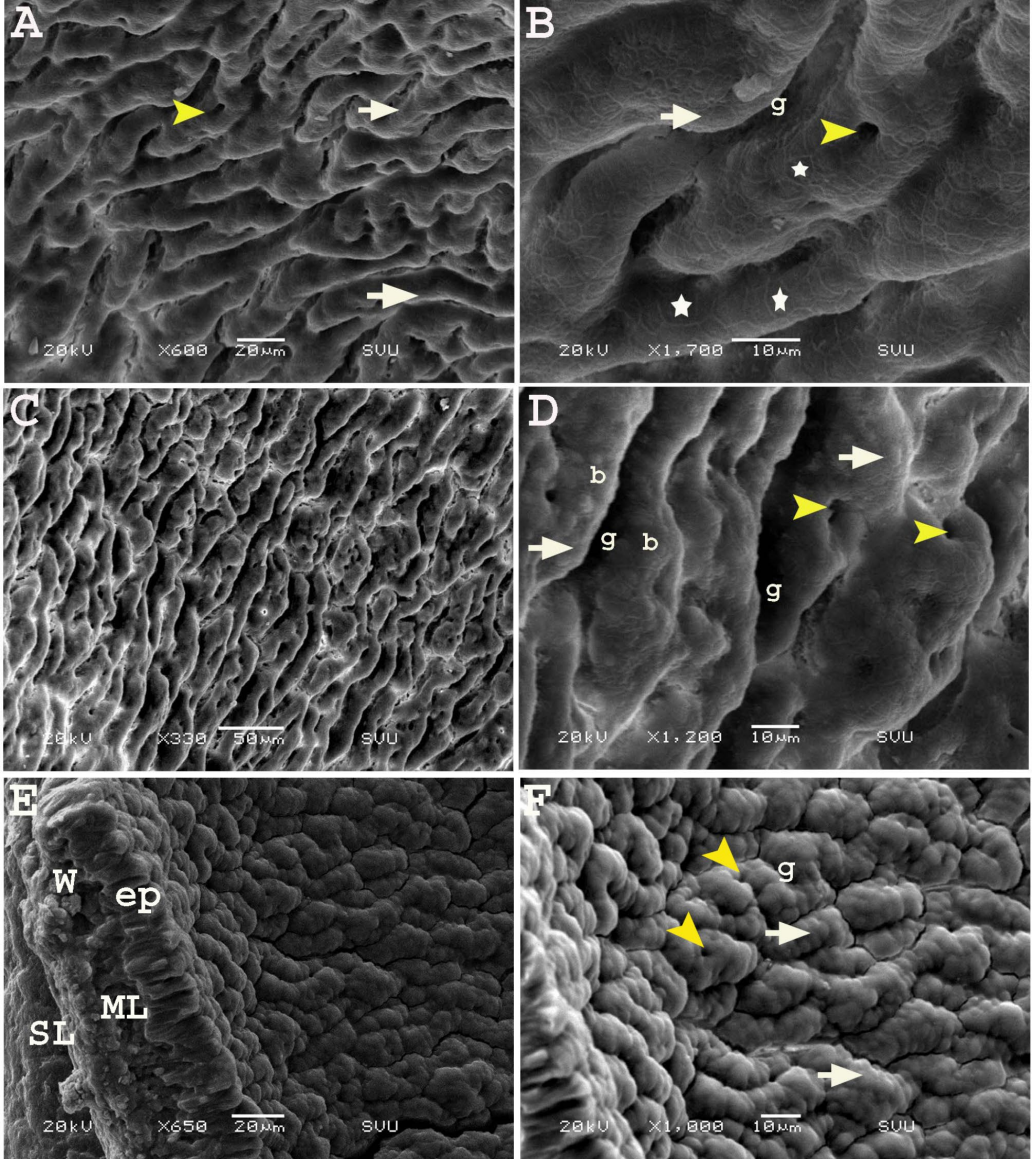
Scanning electron micrograph (SEM) of the colorectum of a quail embryo at day 12 of incubation. Cranial part (**A**, **B**) and caudal part of colorectum (**C-F**). Images B, D and F were details of images A, C and E. The internal surface having elevations (white arrow) and central depression (yellow arrow head), separated with grooves (g), honey-comb (star)and bubbly (b) arrangement of enterocytes, rectal wall (W), epithelium (ep), muscular layer (ML) and serosa (SL)

On the twelfth day of incubation, SEM revealed that the mucosa of the cranial part of the rectum was arranged as irregular elevations (growing villi) with grooves (sulci) resembling a maze or cerebellum surface. The surface of the elevations presented a central depression (groove), and enterocytes were arranged in a hexagonal manner (honeycomb like shape) (Fig. 6A, B). In some areas, the surface elevations had a bubbly appearance instead of a hexagonal shape (Fig. 6C, D). Meanwhile, at the caudal part of the rectum close to its opening in the cloaca, the elevations were more condensed, and the sulci between them were narrower, giving the appearance of the vermis of the cerebellum (Fig. 6E, F).

**Fig. 7:**
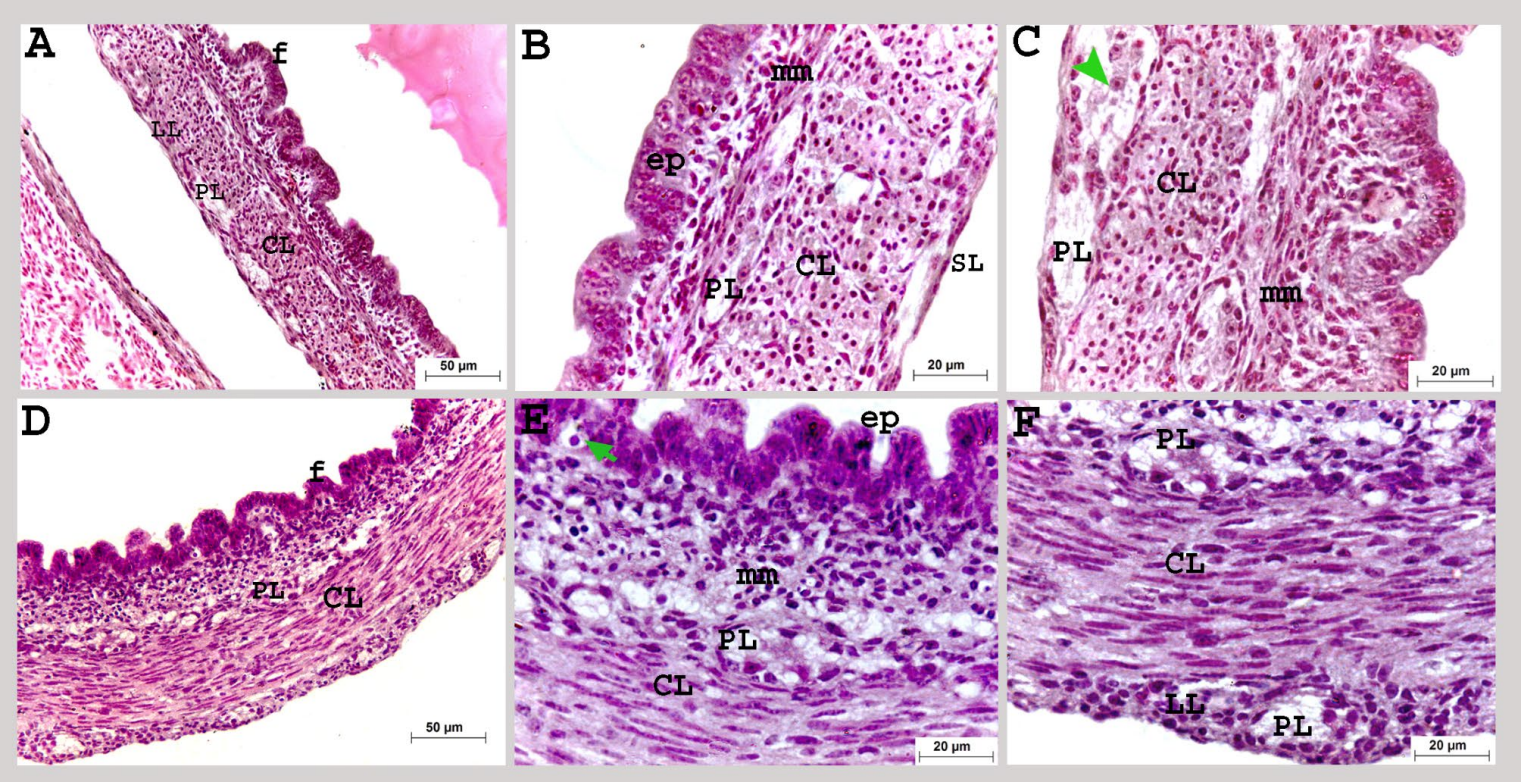
Colorectum of a quail embryo at day 13 (**A-C**) and 15 of incubation (D-F). Histomorphology. Cross paraffin sections (**A-F**). H&E stain (**A-F**). Images B, C, E and Fwere details of images A, D. The colorectal mucosa arranged in folds (f), lined with simple columnar epithelium (ep), enteroendocrine cell (green arrow), muscularis mucosa (mm), circular (CL), longitudinal (LL) muscular layer, myenteric plexus (PL), neurons (green arrow head) and serosa (SL)

On the thirteenth day of incubation, the myenteric plexus was observed at the same level with the outer longitudinal muscular layer (Fig. 7A: C).

On the fifteenth day of incubation, the circular and longitudinal muscular layers continued to develop and increased in thickness, and the submucosal plexus extended as a network beneath the muscularis mucosa (Fig. 7D: F).

**Fig. 8:**
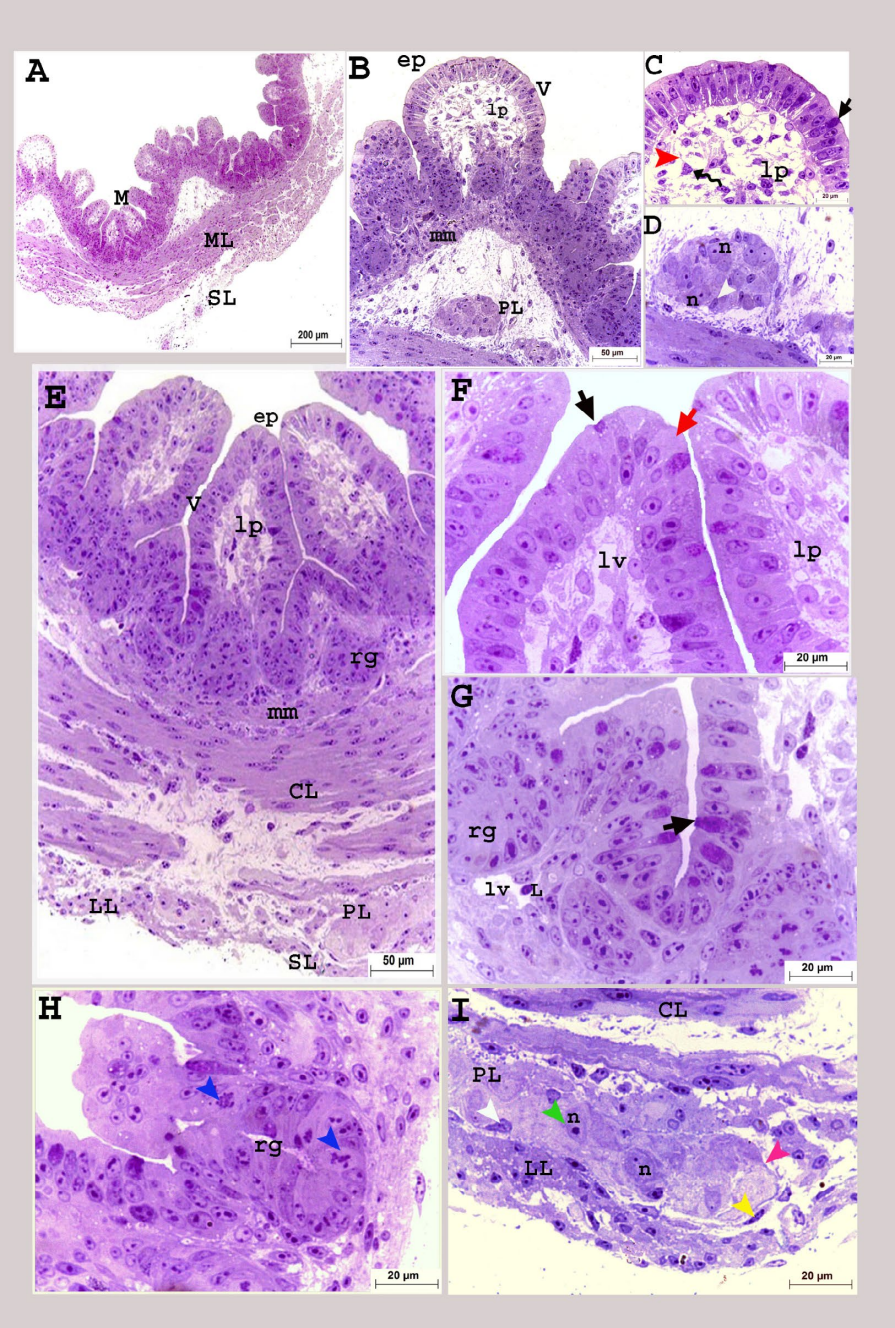
Colorectum of a quail embryo at day 17 of incubation (**A-I**). Histomorphology. Cross semithin sections (**A-I**). Toluidine stain (**A-I**). Images B-I were details of image A. Mucosa (M), muscular layer (ML), simple columnar epithelium (ep), goblet cell (black arrow), enterocyte (red arrow), lamina propria (lp), lymph vessel (lv), lymphocyte (L), villi (V), rectal gland (rg), mitotic divisions (blue arrow head), muscularis mucosa (mm), circular (CL), longitudinal (LL) muscle layers, telocytes (twisted arrows), telopodes (red arrow head), myenteric plexus (PL) consisted of capsule (purple arrow head), contained nerve cell bodies (n and green arrow head), and glial cell (white arrow head), surrounded with flattened cells (yellow arrow head), and serosa (SL). (**A–I**)

**Fig. 9:**
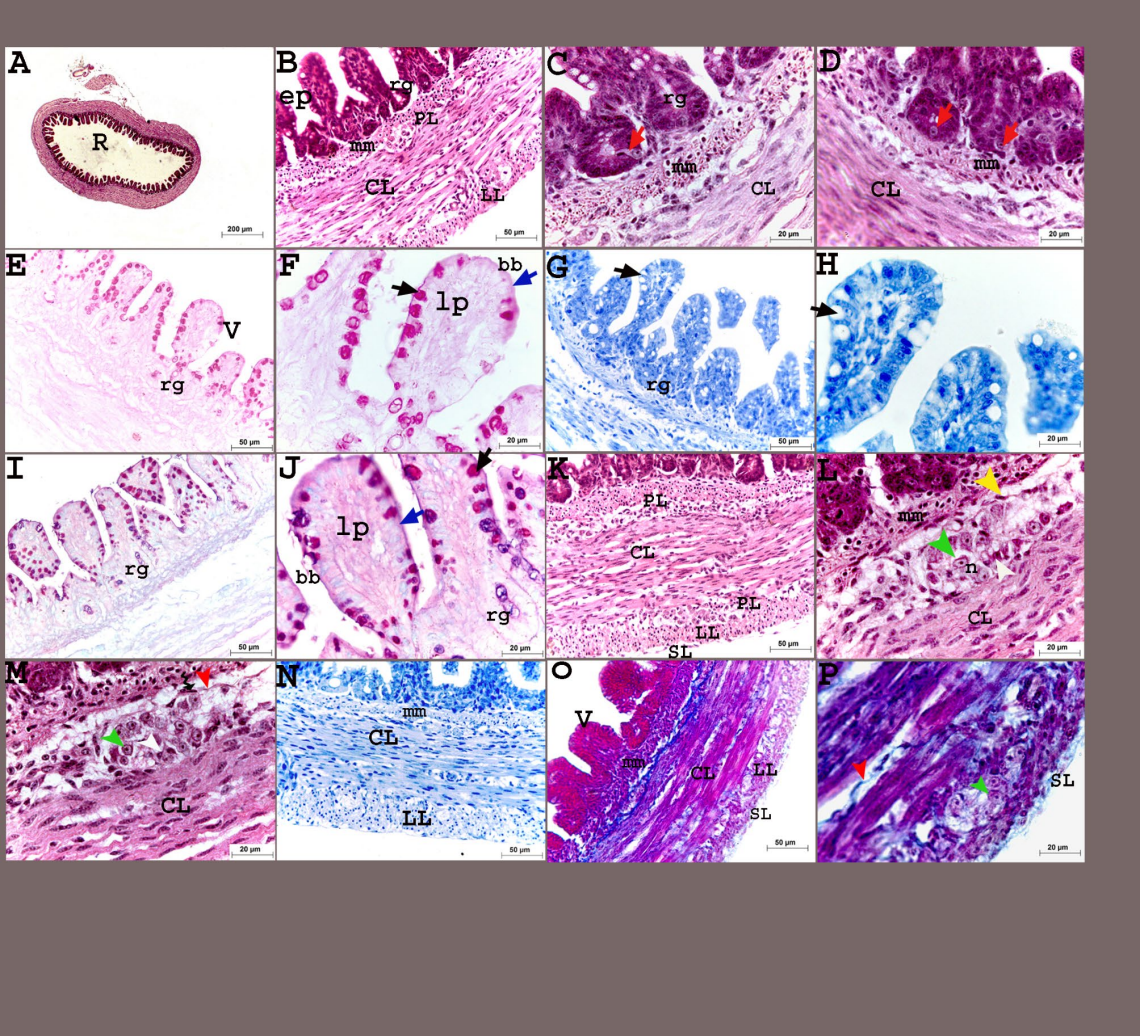
Colorectum of a quail embryo at day 17 of incubation (**A-P**). Histomorphology. Cross paraffin sections (**A-P**). H&E (**A**:**D**, **K**: **M**), PAS (**E**, **F**), AB (**G**, **H**, **N**), PAS and AB (**I**, **J**), and Mallory trichrome stains (**O**, **P**). Images B-P were details of image A. Colorectum (R), simple columnar epithelium (ep), brush border (blue arrow and bb), goblet cell (black arrow), lamina propria (lp), villi (V), rectal gland (rg), muscularis mucosa (mm), circular (CL), longitudinal (LL) muscle layers, myenteric plexus (PL) consisted of nerve cell bodies (n and green arrow head), and glial cell (white arrow head), surrounded with flattened cells (yellow arrow head), telocytes (twisted arrows), telopodes (red arrow head), and serosa (SL)

On the hatching day, the mucosa consisted of a simple columnar epithelium interspersed with goblet cells, lamina propria, and muscularis mucosa. The epithelium and lamina propria folded towards the rectal lumen forming villi and directed downward, forming crypts (rectal glands) (Fig. 8A, B, E). These glands were simple tubular glands with high cellular divisions (Fig. 8G, H). Two different shapes of villi were detected at this age; tongue-shaped and pyramidal-shaped (Figs. 8A, B and E and 9A, B, F and O). The mucosa of the wide cranial rectal portion was more developed and more folded than that of the caudal portion, which enhanced the absorbing surface area (Figs. 8 and 9).

The mucosal epithelium was formed mainly of two types of cells: tall columnar enterocytes and cup-shaped goblet cells filled with metachromatic granules as viewed in the semithin sections. The goblet cells were PAS + giving reddish coloration and reddish bluish coloration when stained in combination with AB but stained negative for AB separately (Figs. 8C, F and G and 9E: J). The lumen of the rectal glands and the brush border of the columnar enterocytes showed positive reaction for acidic and neutral mucins (Fig. 9F, H, J). Another type of cells, known as enteroendocrine cells, was primarily seen in the rectal glands. These cells had triangular shapes with rounded prominent nuclei and faint cytoplasm (Fig. 9C, D).

The muscular tunic was formed of two layers of smooth muscle cells: the inner circular and outer longitudinal muscle layers (Fig. 9K: P). The enteric nervous consisted of two concentric ganglionated plexuses; the myenteric (Auerbach’s) plexus, positioned finally in between the circular and longitudinal muscle layers, and Meissner’s (submucosal) located beneath the muscularis mucosa as groups of neurons and glial cells surrounded with capsule and flattened cells. The serosa was formed of a connective tissue layer encircled by a single layer of flat cells (Figs. 8A, D, E and I and 9B, C, K and L).

**Fig. 10:**
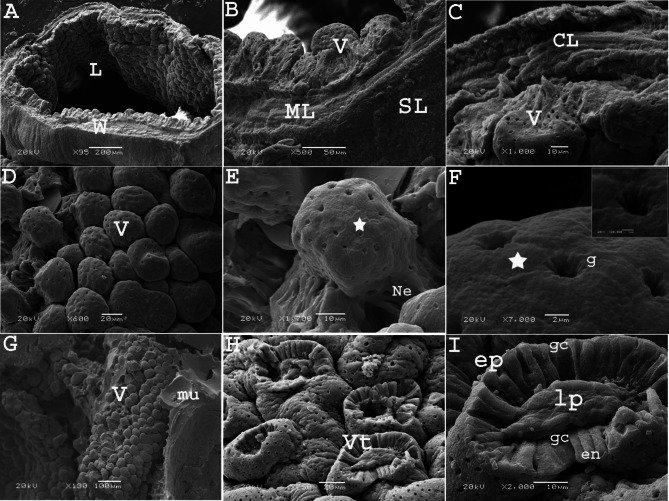
Scanning electron micrographs (SEM) of the colorectum of a quail embryo at day 17 of incubation. Images B, C, E, F, H and I were details of images A, D and G. The rectal wall (W), lumen (L), villi (V), muscular layer (ML), circular muscle layer (CL), serosa (SL), villi neck (Ne) goblet opening (g), honey-comb shaped arrangement of enterocytes (star), villi with losing tips (Vt), mucous secretion (mu), epithelium (ep), goblet cells (gc), lamina propria (lp), and enterocytes (en)

SEM of the rectum on the seventeenth day of incubation revealed that the epithelium was arranged into many villi, similar a mushroom- shape. These villi were also arranged in rows parallel to each other in a regular manner. At high magnification, some villi had a stem (neck) that carried their rounded proximal ends (tips). The outer surface of the villi exhibited circular or oval openings of the goblet cells with irregular outlines (Fig. 10A: F). High magnification of villi with a missing proximal tip showed the arrangement of the lining epithelium cells (enterocytes and goblet cells) in a circular manner around the core of the lamina propria connective tissue resembled the sunflower (Fig. 10G: I).

### Microscopic measurements

Morphometric data clarified that the colorectum had accelerated growth rate throughout the entire incubation period, the diameter, lumen, and villi number increased significantly along the whole incubation period from day 5 to hatching day (Table 1). The colorectal diameter increased sevenfold from day 5 to hatching day (Table 1). The thickness of muscular layer significantly increased on day 12 compared to day 9 (*P* < 0.05). However, there was no significant difference between day 12 and 15, reaching the greatest thickness on hatching day (Table 1). The number of villi was significantly increased from 4 ridges on day 5 to more than one hundred villi at hatching day. The height of villi did not differ between days 5, 9, 12, 15 but increased significantly on the hatching day (Table 1).

### Gross findings and morphometric data

**Fig. 11:**
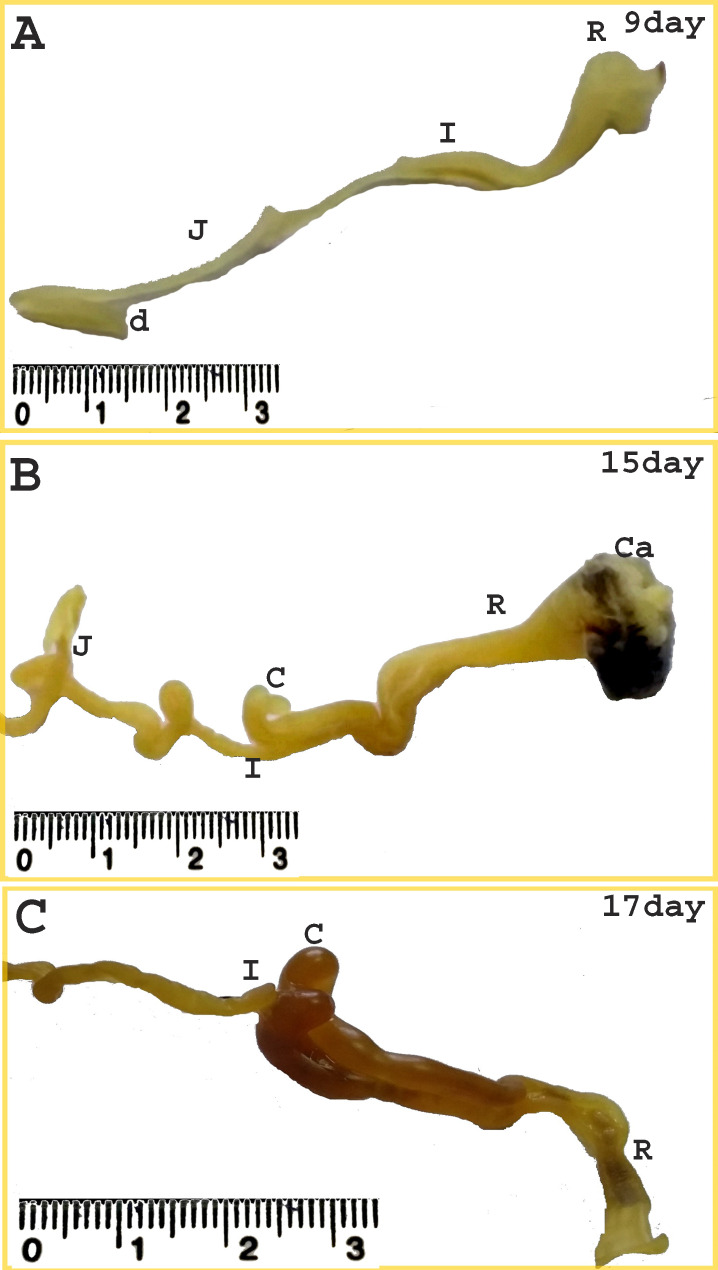
Photographs of the large intestine of a quail embryo at day 9, 15, and 17 of incubation. The duodenum (d), jejunum (J), ileum (I), two caeca (C), colorectum (R), and cloaca (Ca)

**Fig. 12:**
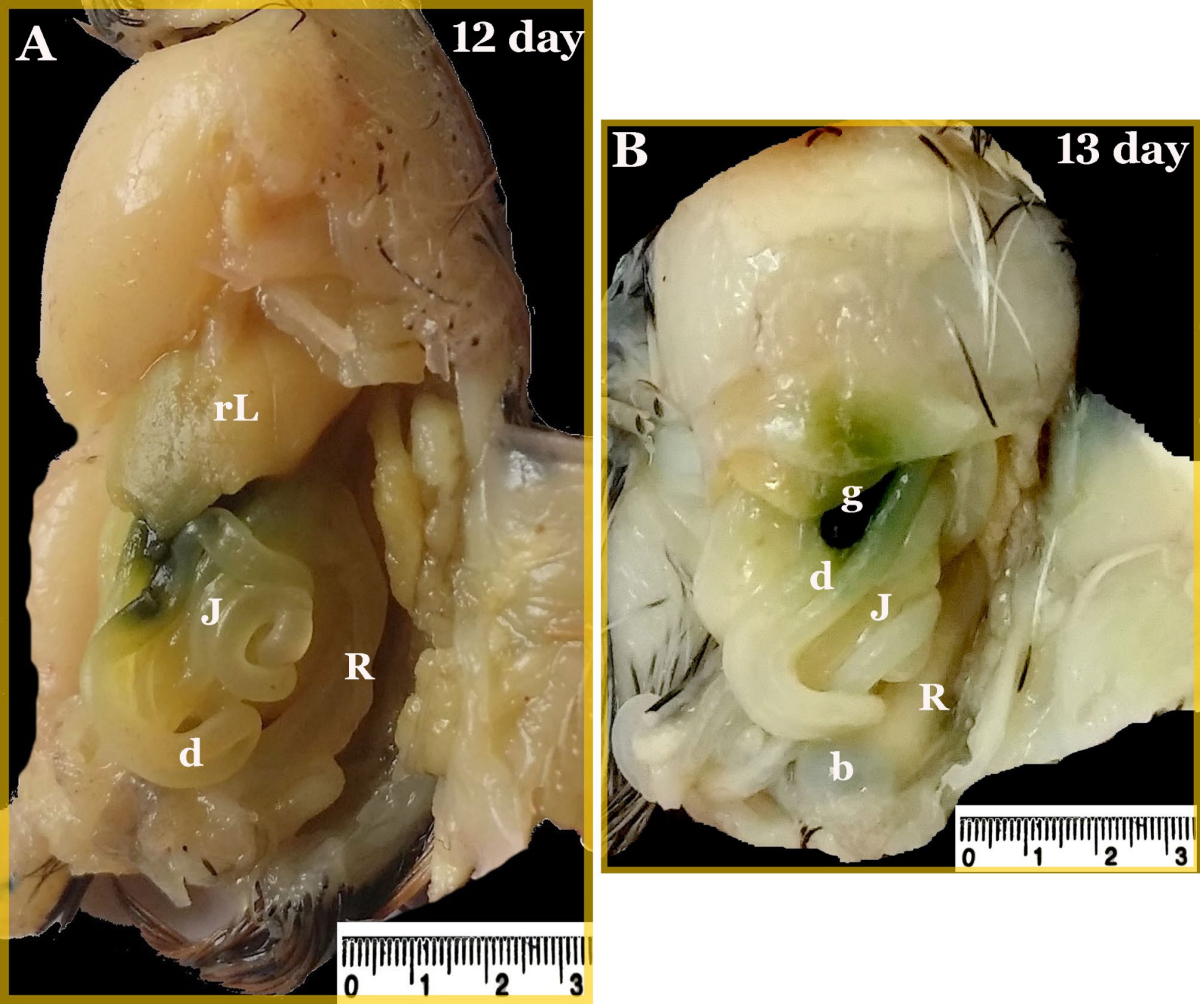
Photographs of the viscera of a quail embryo at day 12 and 13 of incubation. The duodenum (d), jejunum (J), colorectum (R), right liver (rL), gall bladder (g), and bursa (b)

The two main parts of the large intestine of the quail embryo; the two caeca and the colorectum, were almost equally in length during the incubation period reaching a length of 16.36 ± 0.87 and 15.48 ± 0.95 mm, respectively on the hatching day. The colorectum represented approximately 48% of the total length of the large intestine (Fig. 11) (Table 2). On the twelfth and thirteenth day of incubation, the colorectum was recognized as a wide short tube, extended from the junction of the two caeca with the ileum and continued caudally right to the median plane in the body cavity roof. It located dorsal to the gizzard and small intestine, ventral to the primitive kidney, and ended with an expanded end that opened into the cloaca ventral to the bursa (Figs. 11 and 12). All intestinal loops except the colorectum were related to the right surface of the gizzard (Fig. 12B).

## Discussion

The present research focused on the descriptive embryological insights of the colorectum in quail embryos from the 3rd day of incubation to the hatching day and on the role of its morphological function in evolutionary biology.

There is a close relationship between the morphological characteristics and functional features of each biological system, including the GIT [20]. The two caeca and colorectum formed the large intestine of the studied bird. Some scholars found that the large intestine represented only the rectum (twisted tubular organ) such as in zebra finch and starling [2], brown falcon [21], sand grouse and Tauri pigeon [22, 23]. Birds’ rectum is recognized to be involved in the absorption of folic acid [9] and water [24]. It is involved also in the exchange of nitrogen [25], potassium [26], and sodium [27]. Protein digestion is aided by proteolytic bacteria found in the intestinal lumen [28]. Therefore, the variations in the structure and form of the rectal mucosa among avian species will surely reflect the characteristics of these processes [29].

The intestinal tract begins to develop early in fetal life and rapidly continues to grow after delivery. During this period, the intestinal tract undergoes a substantial ontogeny, preparing the newborn for nutrition encounters [30]. According to our recent description [15], the gut tube of quail embryos was distinguished on the third day of incubation but it formed on the fourth day of incubation in the same bird embryo [11]. The rectum represented the caudal part of the hindgut on the 4th day of incubation, which was hung dorsally to the roof of the caudal body cavity with the mesentery and continued ventrally with the cloaca. Extracellular vacuoles were observed within the pseudostratified epithelium of its wall. This finding was also observed by Soliman and Madkour [11], these vacuoles may be interrelated with developmental epithelial cysts.

From the 12th day of incubation to the hatching day, the rectal epithelium was differentiated into villi of simple columnar lining epithelium, and arranged in rows with pyramidal and tongue shapes. These outcomes match with those of Nasrin, et al. [31] and Taki-El-Deen [3]. However, the latter authors reported that leaf-shaped villi occupy the most of the rectal lumen in broiler and winged lapwing. In pheasant and common moorhen, the colorectal villi are short, thick or broad and their other properties are similar to those of the small intestine [1, 32].

Cup-shaped goblet cells were found within the rectal lining epithelium. These cells are principally in charge of producing intestinal mucus. Their numbers progressively increase from the small to large intestine, and the largest numbers are recorded in the colorectum as mentioned in common quail, pheasant, moorhen, and little owl [1, 32–34]. The presence of numerous goblet cells in the rectum with the assistance of its thick muscular layer may facilitate the transit of undigested food to the outside.

It is noteworthy that the studied rectal goblet cells were partially mixed-type (neutral and acidic mucopolysaccharides) with somewhat greater affinity for PAS than for alcian blue stains. Several scholars have also observed the mixed-type goblet cells in the intestine of different birds [32, 35–37]. Scanned rectal samples showed circular or oval goblet cell openings with irregular outlines on the outer surface of the villi.

Undoubtedly, the mucus secretion of the goblet cell plays abundant roles in the intestinal tract: it protects the mucosal surface from invasion by foreign substances, improves the digesta movement and facilitates its absorption, digestibility, and transportation, and provides the energy requirements for the epithelial cells and microvilli for disintegration of the intestinal contents [37, 38]. From our study, the early emergence of goblet cells with acidic mucus production on the hatching day indicates the significance of these cells in protecting the gut mucosa at the beginning of bird feeding.

The rectal epithelium was folded downward, forming rectal glands, and their lumen was positive for PAS and AB which contained both acidic and neutral mucins. Our results are in agreement with those of Taki-El-Deen [3] in the rectal wall of winged lapwing. Our work and that of Shawki [33] in the rock doves confirm that these glands are simple tubular with simple columnar epithelium. The former authors also confirmed the positive PAS and AB reactions for the rectal epithelium. These authors also applied mercuric bromophenol blue stain, which indicates the localization of the protein in the absorptive cells of the mucosal rectal folds.

Telocytes are interstitial cells present in the stroma of many organs, these cells have small bodies with very long prolongations (telopodes) and very thin segments (podomers) with dilated portions (podoms) [39, 40]. Telocytes play a critical role in morphogenetic bioelectrical signaling [41]. Through their secretory ability, they assist in new tissue development by expressing CD34, CD68, and MMP-9 during myogenesis [42]. The current study revealed that on the 6th day, telocytes were small body cells with prolonged telopodes within the lamina propria, forming a network surrounding the circular smooth muscle layer on the 9th day and connecting with them through their telopodes. With age advancement, telocytes were observed around the most rectal wall structures such as lamina propria, muscle layer, blood vessels, and myenteric plexus to assist in the development of these structures. These cells formed a network frame on which the intestinal wall layers were built. Similar observations in the human gastrointestinal tract have been reported by Vannucchi et al. 2013 [43] who added that telocytes express platelet-derived growth factor receptor α (PDGFRα) and form a three-dimensional network in the submucosa and between muscle layers. Telocytes are present around and within muscle bundles, also encircle nerves, blood vessels, fundus of gastric glands, and crypts of the intestine. In Japanese quail, on the hatching day, telocytes are observed in the core of caecal villi, surrounding the crypts of Lieberkühn and muscle layers [44]. Other researchers have shown that telocytes form a subepithelial plexus extending from the stomach to the colon, and secrete Wnt proteins which are important for stem cell proliferation, and epithelial regeneration [45]. They are also detected around the myenteric plexus and in relation to several immunological cells within the intestinal bulb of grass carp, indicating their role in preserving gut immunity [40]. The distribution of subepithelial telocytes in the lamina propria, around and between the muscle layer, blood vessels, and myenteric plexus indicates their potential role in developing these structures and assisting them in their functions.

The gastrointestinal tract is controlled through three regulatory mechanisms; endocrine, paracrine, and intrinsic nervous system (ENS) mechanisms. Overall, neuronic control of colorectal motility occurs through the ENS, which is modified through the extrinsic nervous system, extrinsic sensory innervations, lumbar sympathetic nerves, and sacral parasympathetic nerves [46]. The enteric nervous system (ENS) is composed of a network of neurons and glial cells. These cells originate from the neural crest, then migrate to the gut tube, and eventually encompass the whole length of the gastrointestinal tract, where they control and coordinate different aspects of gastrointestinal function [47]. These cells are arranged in the gut as two ganglionic layers; the Myenteric and Meissner’s ganglia layers [5]. Meissner’s (submucosal) plexus mostly regulates secretion and the flow of blood, while Auerbach’s (myenteric) plexus primarily regulates motility [46]. Consistent with these findings, the results obtained in this study revealed that the ENS started as an aggregation of the ganglionic cells within the mesenchyme of the colorectal wall on day 5 of incubation and the first recognition of the myenteric plexus occurred on day 9 as a group of neurons and glial cells below the inner circular smooth muscle layer, similar to the observations by Uesaka [5] who reported that the submucosal plexus is a subpopulation of cells migrating from the myenteric plexus, and all of these cells originate from vagal neural crest cells. On the other point of view, Abdelhakeem [16] found that both the myenteric and submucosal plexuses develop within the wall of the quail cecum on the twelfth day of incubation as aggregation of neurons bodies. The presence of these plexuses in the rectal wall indicates their role in coordinating the functions of secretions, digestion, absorption, and expulsion of undigested food.

Statistically, our study revealed that the colorectum had accelerated growth, and its length increased from 5.74 ± 0.66 to 15.48 ± 0.95 mm from day 5 to day 17 of incubation. The presence of an occluded rectum was detected on day 4 and the rectum was completely recanalized on the 10th day of incubation. Similarities were detected in the rectum of the chick which measured 20 mm from the fourth day to hatching. The lumen was obliterated on the 6th day and canalized on the 12th day of incubation [48]. Overall, the length of the intestine varies among different avian species in accordance with their dietary habits. Some factors, such as seasonal changes do not effect morphometry [33].

## Conclusion

This study provided sufficient data on colorectal development, including changes in cellular components and the appearance of telocytes and the enteric nervous system (ENS). The current study also revealed that the rectum had accelerated growth, developed craniocaudally and was almost fully developed at hatching. The morphological characteristics of the quail colorectum at different ages were closely related to its functional features.

## Recommendation

Quails should not be fed immediately after hatching based on the study findings, as the colorectum did not completely develop.

## Data Availability

Requests for any data can be made to the corresponding author.
